# Molecular Mechanisms of Floral Boundary Formation in *Arabidopsis*

**DOI:** 10.3390/ijms17030317

**Published:** 2016-03-02

**Authors:** Hongyang Yu, Tengbo Huang

**Affiliations:** 1 College of Life Sciences and Oceanography, Shenzhen University, 3688 Nanhai Ave., Shenzhen 518060, China; hongyangyu@szu.edu.cn; 2 College of Optoelectronic Engineering, Shenzhen University, 3688 Nanhai Ave., Shenzhen 518060, China

**Keywords:** *Arabidopsis*, floral boundary, regulatory pathway, transcription factor, phytohormones

## Abstract

Boundary formation is a crucial developmental process in plant organogenesis. Boundaries separate cells with distinct identities and act as organizing centers to control the development of adjacent organs. In flower development, initiation of floral primordia requires the formation of the meristem-to-organ (M–O) boundaries and floral organ development depends on the establishment of organ-to-organ (O–O) boundaries. Studies in this field have revealed a suite of genes and regulatory pathways controlling floral boundary formation. Many of these genes are transcription factors that interact with phytohormone pathways. This review will focus on the functions and interactions of the genes that play important roles in the floral boundaries and discuss the molecular mechanisms that integrate these regulatory pathways to control the floral boundary formation.

## 1. Introduction

Organ boundaries are groups of specialized cells with restricted growth that are crucial for the development of plants and animals. Boundaries delineate identities by separating distinct functional domains, such as the meristem and organ primordia or adjacent organs, and also function as organizing centers for the downstream signaling events to pattern the organs at later stages [1,2]. Recent studies have uncovered a suite of important genes and pathways that establish and maintain organ boundaries in plant development [2,3,4]. A number of these genes have conserved functions in defining the boundaries of various organs, while others play more specialized roles in a certain patterning events, suggesting a complex regulatory network controlling boundary formation in plants [5,6,7,8,9].

**Figure 1:**
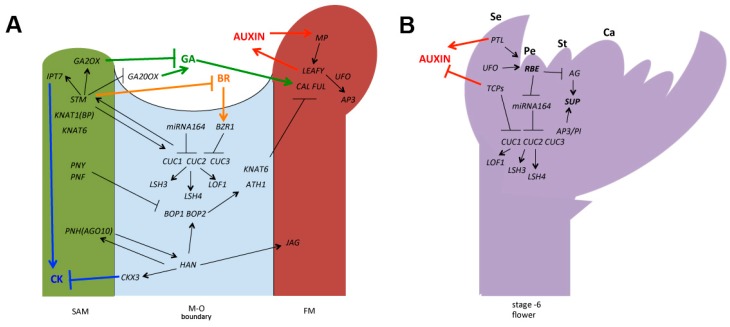
Gene network regulating floral boundaries. (**A**) Major regulatory pathways controlling floral M–O boundaries. Shoot apical meristem is demonstrated in green, M–O boundary is demonstrated in light blue and floral meristem (FM) is demonstrated in brown; (**B**) Key pathways regulating floral O–O boundaries shown in a stage 6 flower. Black arrows and bars represent the positive and negative genetic interactions. Color arrows and bars represent the promoting and repressing controls between genes and phytohormones. Pathways related to the action of phytohormones are indicated as red: auxin; green: Gibberellins (GA); blue: Cytokinins (CK); and orange: Brassinosteroids (BR). In the stage 6 flower, regulatory pathways associated with two floral-specific zinc-finger transcription factors, *RBE* and *SUP*, are highlighted. Se: sepal; Pe: petal; St: stamen; Ca: carpel.

As a key structure of flowering plants, the development of floral organs has been extensively studied [10,11]. Similar to other lateral organs, floral primordia initiate from the shoot apical meristem (SAM) and undergo a complex organogenesis process that incorporates finely controlled cell division, expansion and differentiation [12,13]. Establishment of boundaries is a critical step in floral organogenesis; this includes the formation of boundaries between the meristem and floral organs (the meristem-to-organ boundary; M–O boundary), as well as the boundaries between adjacent floral organs (the organ-to-organ boundary; O–O boundary) [2,3,14]. These boundary regions express a suite of genes with either general or floral-specific functions to define the boundary field and influence the developmental programs of the adjacent floral tissues. Here, we will review the recent advances in the regulation of the floral boundaries in the model species *Arabidopsis thaliana*, particularly the key molecular mechanisms that play specific roles during flower development (as summarized in Figure 1). We will also discuss how these central regulators interact with the internal phytohormone signals to fine-tune the boundary formation in flowers.

## 2. Regulation of the Meristem–Organ (M–O) Boundary Formation in the Flower Development

The *Arabidopsis* shoot apical meristem (SAM) consists of a central zone that stem cells reside in, a peripheral zone that gives rise to organ primordia and a rib zone that forms vascular and interior stem structures [15]. In the reproductive stages, the SAM is converted to an inflorescence meristem where floral meristems arise and further develop to mature flowers [11,16]. In this process, key transcription regulators *LEAFY* (*LFY*) and *APETALA1* (*AP1*) play a crucial role. These floral identity genes are upregulated in response to floral inductive signals, such as the *FLOWERING LOCUS T* (*FT*) pathways, and in turn elicit flowering [11,17,18,19]. Initiation of flowers also requires the formation of the M–O boundary that separates the central zone and peripheral zone in the inflorescence meristem [20,21]. This process is closely related to the local depletion of phytohormone auxin that causes the lower division and growth rate of the M–O boundary cells [2,22]. Consistent with this model, mutants in the auxin efflux transporter gene *PINFORMED1* (*PIN1*) or in the auxin-responsive transcription factor *MONOPTEROS* (*MP*) have naked, “flowerless” inflorescence that is associated with the misexpression of key meristem and organ boundary regulators in the peripheral zone, including *SHOOT MERISTEMLESS* (*STM*), *LEAFY* (*LFY*), and *CUP-SHAPED COTYLEDON* (*CUC*) genes [16,23,24,25,26]. *STM* is a member of the class I KNOTTED1-like (KNOX) genes in the three-amino acid loop extension (TALE) homeodomain protein family and *CUC* genes (*CUC1*, *CUC2* and *CUC3*) are NAC family transcription factors [5,6,7,27]. *STM* and the *CUC* genes are key regulators of meristem and organ boundary development, and play a pivotal role at both vegetative and reproductive stages [28]. *CUC1* and *CUC2* are expressed in the organ boundaries and activate *STM* for meristem initiation [5,29]. *STM* in turn maintains the expression of *CUC* genes in the boundary domains for organ separation; this is likely through the direct regulation of *CUC1* and indirect regulation of *CUC2* and *CUC3* [28].

Other KNOX genes, such as *BREVIPEDICELLUS* (*BP*)/*KNAT1* and *KNAT6*, are also involved in the initiation and maintenance of SAM and organ boundaries in collaboration with *STM* [30,31]. KNOX genes specify meristem and boundary domains in part by modulating the abundance of phytohormones including cytokinins (CK), gibberellins (GA) and brassinosteroids (BR). Maintenance of meristem identity is associated with a high CK to low GA ratio that sustains cell division and inhibits cell differentiation. KNOX genes promote CK levels by activating the CK biosynthetic gene *ISOPENTENYL TRANSFERASE7* (*IPT7*) [32,33], and reduce GA levels by positively regulating GA catabolic GA2-oxidases [34] and negatively regulating GA biosynthetic GA20-oxidases [35]. Brassinosteroids (BR) also play a key role in the formation of organ boundaries. BR-activated *BRASSINAZOLE-RESISTANT1* (*BZR1*) directly represses the expression of *CUC* genes, and lower levels of *BZR1* were observed in the boundary cells [36]. Recent studies in rice showed that KNOX genes down regulate BR signaling in the meristem by direct activation of BR catabolic genes to maintain the SAM activity [37].

These regulatory pathways suggest that a complex interaction between phytohormones and *CUC*/*KNOX* genes controls meristem and boundary formation. These pathways act recurrently at both vegetative and reproductive stages, indicating their conserved roles in the plant life cycle. Conversely, the flower-inducing gene *LFY* specifically regulates organ development in the reproductive phase [19,38]. Auxin signal affects *LFY* through the direct activation of *LFY* by *MP* [39]. *MP* directly activates *LFY* in response to auxin only in the reproductive phase, suggesting a specific molecular function of auxin signaling in reproductive development [39]. *LFY* also directly regulates auxin pathways, which forms a forward-loop to reinforce this relationship [39,40]. In addition to the auxin-associated pathways, *LFY* also regulates flower initiation in response to *PENNYWISE* (*PNY*) and *POUND-FOOLISH* (*PNF*), two BELL-like genes that belong to the same TALE homeodomain-protein family as KNOX genes [41,42,43]. Double mutant *pny pnf* plants do not produce flowers, which is largely due to the overexpression of lateral organ boundary genes *BLADE-ON-PETIOLE1/2* (*BOP1/2*), as well as their downstream genes *KNAT6* and *ARABIDOPSIS THALIANA HOMEOBOX GENE1* (*ATH1*) that also encode TALE homeodomain proteins [44]. The *BOP1/2-KNAT6/ATH1* module blocks flowering via a series of pathways that involves phytohormones Jasmonic acid (JA) and Gibberellins (GA), as well as the microRNA156-*SPL* and-miR172 regulation, to finally effect *LFY* and the other two flower-inducing genes *CAULIFLOWER* (*CAL*) and *FRUITFUL* (*FUL*) [44,45]. Upon flowering, *PNY* and *PNF* negatively regulate *BOP1/*2 and *KNAT6/ATH1*, which in turn activates *LFY*, *CAL* and *FUL* to induce the initiation of flowers [44].

While *LFY* is controlled through boundary-related pathways, it also functions in collaboration with other boundary-regulating genes, such as the F-box protein *UNSUAL FLORAL ORGANS* (*UFO*). *UFO* is specifically expressed in the boundary domain surrounding the *STM*-expressing cells at early floral stages [46,47]. Mutants of *UFO* display a variety of defects including delayed floral meristem development; reduced growth or absence of petals and stamens; and fused floral organs, suggesting a critical role of *UFO* in the regulation of meristem, lateral boundary and organ formation at reproductive stages [48,49,50]. A physical interaction between UFO and LFY recruits the LFY–UFO complex to the promoter of the B-function floral homeotic gene *APETALA3* (*AP3*), and thus activates *AP3* to specify the identity of petals and stamens during flower development [51].

Another key regulator of the floral M–O boundary is the GATA-3 transcriptional factor *HANABA TARANU* (*HAN*) [52,53]. *HAN* is specifically expressed at the floral M-O boundaries and interacts with both the meristem-regulating gene *ARGONAUTE 10*/*PINHEAD* (*PNH*), a founding member of the ARGONAUTE family that acts in small RNA pathways [54,55,56], and the genes required for organ primordia development, including *BOP2* and *JAGGED* (*JAG*), a C2H2 zinc finger transcriptional factor that promotes cell division and growth of lateral organs [53,57,58]. These interactions promote the functions of both meristem and organ primordia-specific genes to delineate these two distinct domains in the shoot apex. In addition, *HAN* also directly activates *CYTOKININ OXIDASE 3* (*CKX3*) to reduce cytokinin levels, which in turn suppresses cell division activity and maintains the M–O boundary [53]. These combined effects suggest that *HAN* plays a pivotal role in the M–O boundary to facilitate communication with the meristem and organ primordia.

## 3. Controlling the Establishment of the Floral Organ–Organ (O–O) Boundary

The *Arabidopsis* flower contains sepals, petals, stamens and carpels arranged in four concentric whorls [11]. Flower development requires the formation of correct O–O boundaries that function to separate adjacent whorls (interwhorl boundaries) and adjacent organs within a whorl (intrawhorl boundaries) [14].

Many genes that establish M–O boundaries also set floral O–O boundaries, such as *BOP1/2* [59,60], *HAN* [52,53] and *CUC* genes [5,6,7]. Among these genes, *CUC*s are probably the earliest ones reported to be implicated in the floral O–O boundary formation. *CUC1*, *CUC2* and *CUC3* are all expressed in the floral organ primordia and boundaries, and the double mutant combinations of the three *CUC* genes form fusions between adjacent floral organs [5,6,61]. More importantly, *CUC* genes are key nodes of the genetic network that regulates floral organ boundaries. They act to control a suite of boundary-regulating genes, including *ORGAN BOUNDARY1* (*OBO1*, also *LIGHT-DEPENDENT SHORT HYPOCOTYLS3*, *LSH3*) and *LSH4* that are members of the ALOG family [62,63], and the Myb-domain transcription factor *LOF1* [36]. *CUC* genes also function downstream of important organ boundary and growth regulators, such as the *TEOSINTE BRANCHED1*, *CYCLOIDEA*, and *PCF* (*TCP*) transcription factors [64,65] and *BZR1* involved in the brassinosteroids (BR) signaling [36].

*CUC1* and *CUC2* are post-transcriptionally regulated by microRNA 164 that is transcribed from three loci: *MIR164a*, *MIR164b* and *MIR164c* [66,67,68,69]. One of the three miRNA164 primary genes, *MIR164c* (also named *EARLY EXTRA PETALS 1*; *EEP1*) plays a floral specific role in petal organogenesis. The mutant in *MIR164c* has extra petals in early-arising flowers that were proposed to be associated with the additional boundary domains formed in the second whorl [67].

The petal-specific function of *EEP1* is controlled in part by the C2H2 zinc finger transcription factor *RABITT EARS* (*RBE*) [8]. *RBE* is specifically expressed in petal primordia and negatively regulates the expression of *MIR164c* by directly interacting with its promoter region [8]*.* The *rbe* mutants exhibit aberrant or absence of petals, and these phenotypes are partly or completely rescued by *eep1*, supporting the model that *MIR164c* acts as a key downstream effector of *RBE* in petal organogenesis [8]*. RBE* also functions non-autonomously in the regulation of sepal boundaries. The fused sepal phenotype in the *rbe* mutant could also be inhibited by *eep1* and enhanced by *cuc1* and *2*, indicating the *miR164-CUC* pathway might be involved in the *RBE*-mediated sepal boundary formation as well [8]. In addition to its regulation of the intra-whorl O–O boundaries, *RBE* also sets the boundary between the second and third whorl by restricting the spatial expression of *AGAMOUS (AG)*, a C-function floral homeotic gene that specifies the identity of stamens and carpels [70]. Another floral organ boundary gene *UFO* seems to act upstream of *RBE* to promote this process, as in the *ufo-11* mutant, *RBE* expression is dramatically down-regulated, while *AG* is ectopically expressed [70].

Besides *UFO*, the tri-helix transcripton factor *PETAL LOSS* (*PTL*) also regulates *RBE*. *PTL* is specifically expressed in the inter-sepal zone and acts in concert with *CUC1* and *CUC2* in the formation of sepal boundaries [9]. Interestingly, *PTL* also controls petal organogenesis in the same pathway with *RBE,* even though *PTL* is not expressed in the petals [71,72]. The regulation of *PTL* on petal organogenesis might be dependent on an interwhorl mobile signal that involves the phytohormone auxin [72], which suggests that the inter-sepal boundary may influence the organ development in the adjacent floral whorl.

Floral organ fusions were also observed in the mutant of F-box gene *HAWAIIAN SKIRT* (*HWS*) [73], which was first reported as the *fused floral organs 1* (*ffo1*) locus in the Landsberg background [73,74]. The loss-of-function *hws-1* resulted in floral organ fusions within a specific whorl (fused sepals and stamens) and also between adjacent whorls (fusion between the third and fourth whorl) [73]. *HWS* may act with *UFO* to regulate organ initiation in the early stages of flower development, as no floral organs were generated in the *hws ufo* double mutant plant [74]. The direct targets of *HWS* are still unknown, but because it encodes an F-box protein, it was proposed that *HWS* might function to degrade growth regulating genes such as those involved in auxin functions [73].

Another floral-specific O–O boundary-regulating gene is *SUPERMAN* (*SUP*), which encodes a C2H2 zinc finger gene closely related to *RBE* [75,76]. *SUP* plays a key role in the establishment of the boundary between the third and fourth floral whorl. Disruption of this boundary in the *sup* mutant leads to the ectopic expression of *AP3* that promotes the formation of extra stamens in the fourth whorl [75,76]. The boundary-specific function of *SUP* is controlled by important floral regulators, including *LFY*, *AP3*, *PISTILATA* (*PI*) and *AG* [77], and *SUP* controls cell proliferations of organ primordia in the third and fourth whorl, in part via regulating auxin and CK-signalling pathways [78]. These interactions suggest that *SUP* is a central gene in the regulatory network of the stamen–carpel boundary that integrates key transcription factors and hormonal regulators of floral organ development.

## 4. Conclusions and Perspectives

The establishment of floral boundaries is critical for the maintenance of inflorescence meristems and the formation of floral organs. Many genes and pathways regulate floral M–O and O–O boundaries; some of them appear to have functions in both vegetative and reproductive development, while others play a more floral-specific role. How these different types of regulators interact in floral boundary formation is one of the important questions that remain to be well understood. Furthermore, many of these boundary genes encode transcription factors or co-factors, suggesting that transcriptomic approaches, such as high-throughput RNA-sequencing and chromatin Immunoprecipiation combined with high-throughput sequencing (ChIP-seq), could unravel the genetic network regulated by these key regulators [79]. In addition, many floral boundary genes function in post-transcriptional regulation, such as the F-box family members. Proteomic studies including yeast-two hybrid screening and co-immunoprecipitation followed by protein-sequencing would be more suitable for identifying the substrates and interacting genes of these post-transcriptional regulators [80,81,82]. A recent study combining boundary-specific gene expression analysis and a genome-wide protein-DNA interaction assay generated an organ boundary-enriched transcriptional network in the *Arabidopsis* leaf development [83]. A similar approach could also be applied in flowers to uncover the pathways that function specifically in floral boundary formation. By comparing the results of these experiments, we could generate a list of common regulators in the overlaps of these datasets. These common genes or pathways likely play pivotal roles in the floral organ boundaries and the relationships of these regulators can form a comprehensive network that explains the molecular mechanism of floral organ boundary formation.

It is also worth noting that a number of the floral boundary regulators crosstalk with phytohormone pathways [2,36,39,84,85]. Techniques for characterizing the biogenesis, transport and response of phytohormones have been largely improved in the past years [86,87]. For instance, the DR5-GUS and DII-VENUS systems applied a visualized auxin sensor in the investigation of the response to auxin [88,89]. Florescent reporters were also employed in the analysis of GA and BR in plants [90,91]. Application of these molecular tools in boundary-specific assays will help elucidate the spatial and temporal regulation of phytohormone signaling in the boundary field and better understand how hormonal pathways and other boundary regulators are coordinated in the formation of floral boundaries. We expect that the integration of the information from all these approaches will provide us with a complex regulatory map that not only shows the details of gene functions and interactions in the floral boundaries, but also directs us to further explore the novel molecular mechanisms underlining the boundary formation in flower development.
