# Correction: Yu, H.; et al. Molecular Mechanisms of Floral Boundary Formation in Arabidopsis. *Int. J. Mol. Sci.* 2016, *17*, 317

**DOI:** 10.3390/ijms19071845

**Published:** 2018-06-22

**Authors:** Hongyang Yu, Tengbo Huang

**Affiliations:** 1 College of Life Sciences and Oceanography, Shenzhen University, 3688 Nanhai Ave., Shenzhen 518060, China; hongyangyu@szu.edu.cn; 2 Key Laboratory of Optoelectronic Devices and Systems of Ministry of Education and Guangdong Province, College of Optoelectronic Engineering, Shenzhen University, 3688 Nanhai Ave., Shenzhen 518060, China

The authors wish to make the following correction to their paper [1].

We would like to change the second affiliation on Page 1 of our paper from “College of Optoelectronic Engineering, Shenzhen University, 3688 Nanhai Ave., Shenzhen 518060, China” to “Key Laboratory of Optoelectronic Devices and Systems of Ministry of Education and Guangdong Province, College of Optoelectronic Engineering, Shenzhen University, Shenzhen 518060, China”.

These changes have no material impact on the conclusions of our paper. The authors apologize for any inconvenience caused to the readers by these changes. The manuscript will be updated and the original will remain online on the article webpage, with a reference to this Correction.
